# A Two-Layer, Energy-Efficient Approach for Joint Power Control and Uplink–Downlink Channel Allocation in D2D Communication

**DOI:** 10.3390/s20113285

**Published:** 2020-06-09

**Authors:** Li Zhou, Yucheng Wu, Haifei Yu

**Affiliations:** 1 School of Microelectronics and Communication Engineering, Chongqing University, Chongqing 400044, China; zhoulipower@cqu.edu.cn (L.Z.); yuhaifei@cqu.edu.cn (H.Y.); 2 State Key Laboratory of Power Transmission Equipment & System Security and New Technology, Chongqing 400044, China

**Keywords:** D2D communication, cellular networks, energy-efficiency, resource allocation

## Abstract

Energy efficiency (EE) is a critical performance indicator for the device-to-device (D2D) communication underlaying cellular networks due to limited battery capacity and serious interference between user equipment. In this study, we proposed a power control and channel allocation scheme for the EE maximization of the D2D pairs, while jointly reusing uplink–downlink resources and guaranteeing the cellular users’ (CUs) quality of service (QoS). The formulated problem was a mixed-integer nonlinear programming (MINLP) problem, which is generally an unsolved non-deterministic polynomial-time hardness (NP-hard) problem within polynomial time. To make it tractable to solve, the original problem was divided into two sub-problems: power control and channel allocation. A power control algorithm based on the Lambert W function was proposed to maximize the EE of the individual D2D pair. Assigning either an uplink or downlink resource to reuse, the EE of each D2D pair was calculated using the power control results. A channel allocation scheme based on the Kuhn–Munkres algorithm utilized the EE weights to optimize the overall EE of the D2D pairs. The simulation results verified the theoretical analysis and proved that the proposed algorithm could remarkably improve the EE of D2D pairs while guaranteeing the QoS of the CUs.

## 1. Introduction

With the rapid development of the Internet of things (IoT) and cellular technology, the demand for higher data rates and radio spectrum resources has continued to increase over the past decade [1,2]. As one of the critical technologies of 5G [3], device-to-device (D2D) communication is a short-distance, low-power communication technology that enables dialogue using direct links and omnipresent information interaction [4,5]. Underlaying general cellular networks, D2D communication brings multiple benefits to network capacity and significant performance improvements to the user experience [6,7].

Due to the limited spectrum and battery capacity, D2D pairs share the same spectrum with cellular users (CUs) by taking advantage of proximity and reuse gains [8]. Maximizing the spectrum efficiency (SE) through sophisticated resource allocation is a primary method used for this. Lei et al. [9] investigated resource allocation problems by considering queuing models and delay constraints. According to issues in different situations, including multi-layer cellular networks [10], relay-assisted transmission [11], and video communication networks [12], resource allocation algorithms were proposed to optimize performance through spectrum reuse.

All the above studies focus on improving the significant SE performance to design higher-capacity wireless systems. However, the energy consumption of user equipment is increasing to meet the growing demand from applications, which in turn leads to a rapid increase in energy consumption and more interference between equipment [13]. Additionally, due to the slow improvement in battery technology, the gap between the required energy consumption and the available battery capacity has grown exponentially. Therefore, the design of D2D communication systems with energy efficiency (EE) [14] as a performance indicator has recently attracted widespread attention in various fields.

The energy consumption of a whole communication process includes computing [15], storage [16], and transmission [17]. We pay more attention to the last one, namely the extensive transmission energy consumption. An energy-efficient content transmission system was proposed in Zhou et al. [18] to realize the large-scale content transmission among mobile devices. Considering the D2D pairs as bidders and the cellular network as the auctioneer, an auction-based power allocation, channel selection, and cooperative relay selection algorithm for EE optimization was proposed in Wang et al. [19]. Under the constraints of the minimum user data rate and the fixed bandwidth allocated to the D2D link, the network throughput was maximized in Chen et al. [20]. By transforming fractional programming into a solvable problem, Jiang et al. [21] combined the iterative resource allocation and power control to maximize the EE. Wei et al. [22] demonstrated the tradeoff between EE and SE through theoretical analysis. Aiming at reducing energy consumption and interference, Li et al. [23] proposed a novel socially aware, energy-efficient relay selection scheme based on game theory. Most of the above works consider the matching problem between the D2D pairs and the CUs. However, the performance of the CUs is seldom considered during the optimization process.

With channel gain information, a greedy heuristic algorithm was proposed in Zulhasnine et al. [24] to control the interference between user equipment. Li et al. [25] addressed an alliance formation game theory method that converges to a Nash-stable equilibrium, archiving maximization of the sum rate. Compared with direct D2D communication and traditional cellular communication via base stations (BSs), Wei al. [22] researched the multi-hop D2D communication scenario. Combining the channel characterization of optimal power and a graph-based channel allocation algorithm, Hoang et al. [26] maximized the weighted system sum rate. Most of the above works aimed to optimize the overall performance, with good results. However, the performance improvement of D2D pairs is seldom considered.

In recent years, there have also been some effective solutions to D2D resource allocation problems. Kaufman et al. [27] developed a distributed dynamic spectrum protocol that achieved valid power savings through a single-hop or multi-hop route establishment. Wang et al. [28] studied a novel distributed game source selection and power control scheme, which improved transmission quality with latency constraints. While meeting the requirements of the D2D pairs and CUs, Rahman et al. [29] optimized a power allocation solution for D2D sources and D2D relays in terms of maximizing the EE. Most of the above works mainly focused on the power control for reducing interference in the resource allocation of D2D communication. However, joint channel allocation and power control for the maximization of system performance have seldom been considered.

The D2D communication network architecture should meet the quality of service (QoS) requirements of the CUs as well as solve EE optimization problems [14]. Liu et al. [30] focused on the EE maximization problem with the QoS constraints of both the CUs and D2D pairs in mind. After the transmission power is allocated, game theory is utilized to establish the preferences of each user’s equipment and obtains a stable matching result. Kai et al. [13] designed a joint uplink subcarrier assignment and power allocation to minimize power consumption. An iterative non-cooperative power game with the Gale–Shapley algorithm was proposed to optimize EE in Zhou et al. [31], which was extended to a context-aware partner selection of D2D pairs in Zhou et al. [32]. A joint resource allocation scheme was investigated in Zhang et al. [33] for downlink systems, which could obtain a near-optimal solution with low computational complexity. However, the above works were mainly based on the assumption that only uplink or downlink spectrum resources could be shared. Hence, the performance could be further improved as the spectrum resources have not been fully utilized.

Motivated by the aforementioned review, we formulated a power control and channel allocation problem for the EE maximization of D2D pairs, while jointly reusing uplink–downlink resources and guaranteeing the QoS of the CUs.

The main contributions of this paper are summarized as follows:

1. This study derived a problem formulation for optimizing the achievable EE of D2D pairs under uplink–downlink resources reuse, transmission power, and QoS constraints. The formulation obtained was a mixed-integer nonlinear programming (MINLP) problem, which is generally an unsolved non-deterministic polynomial-time hardness (NP-hard) problem within polynomial time [31,34]. To make it tractable to solve, the original problem was transformed into two sub-problems.
2. One main focus of this study was to derive a closed-form expression of power allocation for maximizing the EE of an individual D2D pair while satisfying the QoS of the CUs and D2D pairs. Taking into account reusing uplink–downlink resources, we modeled the power allocation problem as an equivalent convex optimization. The optimal transmission power was further obtained based on the Lambert W function [35].
3. Finally, based on the Kuhn–Munkres [36] algorithm, a channel allocation scheme was designed to optimize the overall EE of D2D pairs through the power control results. The simulation results verified the theoretical analysis and demonstrated that the proposed algorithm obtained remarkable EE performance gains and performed better than existing algorithms.

The remaining parts of this paper are outlined as follows. Section 2 provides the system model and a detailed description of the objective function. Section 3 develops the power allocation algorithm based on the Lambert W function and the channel-matching algorithm based on the Kuhn–Munkres algorithm. Simulation results and future research directions are presented and discussed in Section 4. The conclusion is summarized in Section 5.

## 2. System Model and Problem Formulation

In this section, we first provide a detailed description of a one-to-one system model of the D2D communications underlaying cellular networks. Then, the formulation of the energy-efficient resource allocation problem is presented. The notation list in Appendix A summarizes the following main variables and parameters used in this study.

### 2.1. System Model

**Figure 1:**
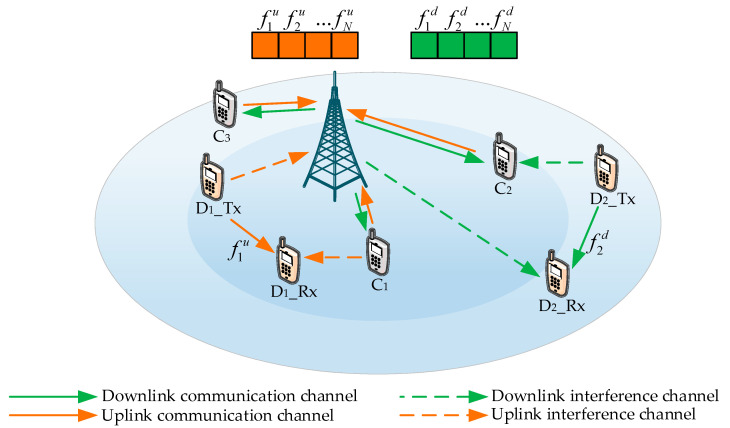
Device-to-device (D2D) communication in a one-to-one reusing scenario diagram.

Figure 1 shows a single-cell network model for frequency division duplex (FDD) communication, which jointly reuses uplink–downlink resources. There are N CUs represented by set $C=\{C_{1},...,C_{n},...,C_{N}\}$, and M D2D pairs represented by set $D=\{D_{1},...,D_{m},...,D_{M}\}$. There are two kinds of communication in this scenario: (1) the traditional communication between the BS and the CUs, and (2) D2D direct communication. Each CU is assigned one orthogonal uplink channel and one orthogonal downlink channel. The D2D pairs reuse the CU’s resources in an underlaying mode. Each D2D pair can only reuse at most one CU’s resource, and each CU’s resource can only be reused by at most one D2D pair. To reduce the complexity of the modulation and demodulation, each D2D pair can either reuse an uplink resource or downlink resource.

We denote $f_{n}^{u}$ as the uplink resource and $f_{n}^{d}$ as the downlink resource of the $C_{n}$. The CUs do not interfere with each other since the uplink resources $f_{1}^{u},f_{2}^{u},...,f_{N}^{u}$ and downlink resources $f_{1}^{d},f_{2}^{d},...,f_{N}^{d}$ are orthogonal. As illustrated in Figure 1, the D2D user $D_{1}\_\mathrm{Tx}$ selects the uplink resource $f_{1}^{u}$ and $D_{2}\_\mathrm{Tx}$ selects the downlink resource $f_{2}^{d}$, which aims at improving the overall EE of the D2D pairs. Therefore, the D2D user $D_{1}\_\mathrm{Rx}$ is interfered by the CUs, and the D2D user $D_{2}\_\mathrm{Rx}$ is interfered by the BS. Similarly, the CUs and BS also suffer from interferences, which are shown in Figure 1.

Considering the effects of multipath fading and shadow fading, this study utilized the path loss model in Kaufman et al. [27], and the interference channel gain from $C_{n}$ to the D2D receiver of $D_{m}$ can be expressed as:

(1) $$g_{C_{n.m}}=K\beta_{n,m}\lambda_{n,m}d_{n,m}^{-\alpha },$$

where K represents the pathloss constant, $\beta_{n,m}$ represents the multipath fading parameter from $C_{n}$ to receiver of $D_{m}$ with an exponential distribution, $\lambda_{n,m}$ represents the shadow gain from $C_{n}$ to the receiver of $D_{m}$ with a log-normal distribution, $d_{n,m}$ indicates the distance from $C_{n}$ to the receiver of $D_{m}$, and α indicates the pathloss factor. Similarly, the channel gain of $D_{m}$ is expressed as $g_{D_{m,m}}$. The channel gain between $C_{n}$ and the BS is expressed as $g_{C_{n,B}}$. The interference channel gain from the D2D transmitter of $D_{m}$ to the BS is expressed as $g_{D_{m,B}}$. The interference channel gain from the BS to the D2D receiver of $D_{m}$ is expressed as $g_{C_{B,m}^{}}$. The interference channel gain from the D2D transmitter of $D_{m}$ to $C_{n}$ is expressed as $g_{D_{m,n}^{}}$.

This study assumed that the four kinds of link information in the network can be obtained by the BS, including the link information between the CU and the BS, the link information between the D2D pair and the BS, the link information between the D2D pair and the other D2D pair, and the link information from the CU to the D2D pair. That is, the BS has the perception function of all link channel information. How the BS obtains the link information between users is not the focus of this study.

### 2.2. Problem Formulation

After formulating a one-to-one system model of the D2D communications, the problem to be solved is described as follows: when the admissible D2D pairs reuse uplink resources or downlink resources of the CUs in an underlaying mode, the problem involves maximizing the achievable EE of the D2D pairs while satisfying the transmission power and QoS constraints of the D2D pairs and CUs.

Based on the system model of Figure 1, we assumed that each admissible D2D pair can only reuse at most one CU’s resource, and each CU’s resource can only be reused by at most one D2D pair. First, we considered the scenario where D2D pairs reuse the uplink channel resources of the CUs. The signal received by the BS includes not only the communication signal from CUs but also the interference signal from the D2D pairs. The received signal at the BS is expressed as:

(2) $$z_{n}^{rx}=\sqrt{p_{n}^{}}g_{C_{n,B}}x_{n}+\sqrt{p_{m}^{u}}g_{D_{m,B}}y_{m}+\zeta_{n},$$

where $p_{n}^{}$ and $p_{m}^{u}$ represent the transmission power of $C_{n}$ and the transmission power of the $D_{m}$ reusing uplink channel, respectively. $x_{n}$ and $y_{m}$ represent the transmission signal of $C_{n}$ and the transmission signal of $D_{m}$, respectively. $\zeta_{n}$ represents the noise in each channel, which was assumed to be Gaussian white noise with a mean of zero and a power of $\delta^{2}$.

We defined binary variables $\chi_{m,n}^{u}$ that represent the scenario where the uplink channel resource $f_{n}^{u}$ of $C_{n}$ is allocated to $D_{m}$, and then we set $\chi_{m,n}^{u}=1$, otherwise $\chi_{m,n}^{u}=0$. When $\chi_{m,n}^{u}=1$, there is signal interference between two users’ equipment and the expression of the signal-to-interference-plus-noise ratio (SINR) at the BS is as follows:

(3) $$\gamma_{n}^{u}=\frac{p_{n}^{}g_{C_{n,B}}}{\delta^{2}+\chi_{m,n}^{u}p_{m}^{u}g_{D_{m,B}}}.$$

When the D2D pair $D_{m}$ does not reuse the uplink channel resource $f_{n}^{u}$ of $C_{n}$, then $\chi_{m,n}^{u}=0$. Hence, the SINR at the BS reaches the maximum value, which is expressed as $\gamma_{n}^{u}=\frac{p_{n}^{}g_{C_{n,B}}}{\delta^{2}}$.

The received signal at the D2D pair $D_{m}$ contains three parts, which are the D2D communication signal, the interference signal from the CU, and the channel noise. Therefore, the received signal at the D2D pair $D_{m}$ is:

(4) $$y_{m}^{rx}=\sqrt{p_{n}^{}}g_{C_{n,m}}x_{n}+\sqrt{p_{m}^{u}}g_{D_{m,m}}y_{m}+\zeta_{m}.$$

Consequently, when the D2D pair $D_{m}$ reuses the uplink channel resource $f_{n}^{u}$ of $C_{n}$, the SINR at the receiver of the D2D pair is:

(5) $$\gamma_{m}^{u}=\frac{p_{m}^{u}g_{D_{m,m}}}{\delta^{2}+p_{n}^{}g_{C_{n,m}}}.$$

Similarly, when the D2D pair $D_{m}$ reuses the downlink channel resource $f_{n}^{d}$ of $C_{n}$, we obtain that the SINR of the receiver of $C_{n}$ is $\gamma_{n}^{d}$ and the SINR at the receiver of the D2D pair is $\gamma_{m}^{d}$. The expressions are respectively expressed as:

(6) $$\gamma_{n}^{d}=\frac{p_{B}^{}g_{C_{n,B}}}{\delta^{2}+\chi_{m,n}^{d}p_{m}^{d}g_{D_{m,n}^{}}},$$

(7) $$\gamma_{m}^{d}=\frac{p_{m}^{d}g_{D_{m,m}}}{\delta^{2}+p_{B}^{}g_{C_{B,m}^{}}},$$

where $p_{B}^{}$ and $p_{m}^{d}$ represent the transmission power of BS and the transmission power of the $D_{m}$ reusing downlink channel, respectively. $\chi_{m,n}^{d}$ represents the scenario where the downlink resource $f_{n}^{d}$ of $C_{n}$ is allocated to $D_{m}$, and then we set $\chi_{m,n}^{d}=1$, otherwise $\chi_{m,n}^{d}=0$.

From Equations (5) and (7), the SE (defined as bits/s/Hz) of the $D_{m}$ reusing uplink channel of $C_{n}$ is:

(8) $$R_{m,n}^{u}=\log_{2}(1+\gamma_{m}^{u})=\log_{2}(1+\frac{p_{m}^{u}g_{D_{m,m}}}{\delta^{2}+p_{n}^{}g_{C_{n,m}}}).$$

The SE of the $D_{m}$ reusing the downlink channel of $C_{n}$ is:

(9) $$R_{m,n}^{d}=\log_{2}(1+\gamma_{m}^{d})=\log_{2}(1+\frac{p_{m}^{d}g_{D_{m,m}}}{\delta^{2}+p_{B}^{}g_{C_{B,m}^{}}}).$$

Generally, the definition of EE (bits/J/Hz) is the ratio of total SE (bits/s/Hz) to the total energy consumption (W) [37], where the total SE of the D2D pairs is equal to the total SE of all the D2D pairs accessing the network. We set $P_{0}$ to represent the circuit power consumption of a single device. Therefore, the total energy consumption of the D2D pairs is equal to the total energy consumption of the devices, which is expressed as $\sum_{m=1}^{M}\sum_{n=1}^{N}\left[\chi_{m,n}^{d}p_{m}^{d}+\chi_{m,n}^{u}p_{m}^{u}\right]+\sum_{m=1}^{M}2P_{0}$. The total EE of the D2D pairs can be expressed as:

(10) $$\eta_{ee}=\frac{\sum_{m=1}^{M}\sum_{n=1}^{N}\left[\chi_{m,n}^{d}R_{m,n}^{d}+\chi_{m,n}^{u}R_{m,n}^{u}\right]}{\sum_{m=1}^{M}\sum_{n=1}^{N}\left[\chi_{m,n}^{d}p_{m}^{d}+\chi_{m,n}^{u}p_{m}^{u}\right]+\sum_{m=1}^{M}2P_{0}}.$$

To maximize the EE of the D2D pairs while meeting the QoS requirements of the D2D pairs and CUs, the objective function of the optimization problem can be formulated as:

(11) $$\eta_{ee}=\underset{p_{m}^{u},p_{m}^{d},p_{n}^{},p_{B}^{},\chi_{m,n}^{d},\chi_{m,n}^{u}}{\max}\frac{\sum_{m=1}^{M}\sum_{n=1}^{N}\left[\chi_{m,n}^{d}R_{m,n}^{d}+\chi_{m,n}^{u}R_{m,n}^{u}\right]}{\sum_{m=1}^{M}\sum_{n=1}^{N}\left[\chi_{m,n}^{d}p_{m}^{d}+\chi_{m,n}^{u}p_{m}^{u}\right]+\sum_{m=1}^{M}2P_{0}},$$

(12) $$\sum_{m}[\chi_{m,n}^{u}+\chi_{m,n}^{d}]\leq 1,\chi_{m,n}^{u},\chi_{m,n}^{d}\in \{0,1\},\forall D_{m}\in D_{A},\forall C_{n}\in C,$$

(13) $$\sum_{n}[\chi_{m,n}^{u}+\chi_{m,n}^{d}]\leq 1,\chi_{m,n}^{u},\chi_{m,n}^{d}\in \{0,1\},\forall D_{m}\in D_{A},\forall C_{n}\in C,$$

(14) $$0\leq p_{n}^{}\leq p_{c}^{max},\forall C_{n}\in C,$$

(15) $$0\leq p_{m}^{u}\leq p_{d}^{max},0\leq p_{m}^{d}\leq p_{d}^{max},\forall D_{m}\in D_{A},$$

(16) $$0\leq p_{B}^{}\leq p_{B}^{max},\forall C_{n}\in C,$$

(17) $$\gamma_{n}^{u}\geq \xi_{min}^{CUs},\gamma_{n}^{d}\geq \xi_{min}^{CUs},\forall C_{n}\in C,$$

(18) $$\gamma_{m}^{u}\geq \xi_{min}^{D2D},\gamma_{m}^{d}\geq \xi_{min}^{D2D},\forall D_{m}\in D_{A},$$

where $p_{d}^{max}$, $p_{c}^{max}$, and $p_{B}^{max}$ represent the maximum transmission power of the D2D pairs, CUs, and BS, respectively. $\xi_{min}^{CUs}$ and $\xi_{min}^{D2D}$ are the minimum SINR thresholds for the CUs and D2D pairs, respectively.

In Equation (11), the transmission power $\left(p_{n}^{}\;,\;p_{B}^{}\;,\;p_{m}^{d}\;,\;p_{m}^{u}\right)$ and the binary variables of the channel allocation $\left(\chi_{m,n}^{u},\chi_{m,n}^{d}\right)$ are the optimization variables. Meanwhile, the EE of the D2D pairs is the optimization goal. Inequalities (12) to (18) are the constraint conditions of this optimization problem, which specifies the QoS requirements of the D2D pairs and CUs. $D_{A}(D_{A}\subseteq D)$ represents a set of admissible D2D pairs, which satisfies the QoS constraint conditions of the D2D pairs and CUs.

Constraints (12) and (13) indicate the constraints stating that at most one channel resource can be reused simultaneously by one admissible D2D pair and one CU. Furthermore, each D2D pair can either reuse a CU’s uplink resource or downlink resource. Hence, each D2D pair will choose its reusing mode according to the EE of its different reusing mode. Constraints (14), (15), and (16) ensure that the power allocations of the CU, D2D pairs, and BS do not exceed their respective maximum allowed transmission power. Constraint (17) specifies the QoS requirement stating that the SINR of the CUs does not fall below minimum SINR thresholds. Similarly, Constraint (18) represents the minimum SINR requirement of the D2D pairs.

By observing the objective Equation (11), we found that the formulation can be considered an MINLP problem, which contains integer variables $\left(\chi_{m,n}^{u},\chi_{m,n}^{d}\right)$. Thus, the formulation obtained is generally an unsolved NP-hard problem within polynomial time. To solve the MINLP problem, we converted the original problem into two sub-problems and solved it in a tractable manner. Sub-problem 1 aimed to maximize the EE of an individual D2D pair while jointly reusing uplink–downlink resources and guaranteeing the QoS requirements. Sub-problem 2 aimed at further optimizing the overall EE of the D2D pairs through channel allocation.

## 3. Resource Allocation Algorithm for Maximizing EE

In this section, we introduce the proposed two-layer energy-efficient approach. Taking into account reusing uplink–downlink resources, we decompose the original MINLP problem into two sub-problems: optimal power control and channel allocation. First, under the QoS constraints of the CUs and D2D pairs, the transmission power of each D2D pair and CU is derived based on the Lambert W function in Section 3.1. Then, taking advantage of the optimized power control results, the channel allocation scheme of the D2D pairs and CUs based on the Kuhn–Munkres algorithm is presented in Section 3.2.

### 3.1. Power Control

This subsection mainly focuses on the sub-problem of transmission power control, which aimed to maximize the EE of an individual D2D pair. When a single D2D pair reuses uplink or downlink channel resources, according to whether user equipment satisfies QoS requirements, the access state of the D2D pair is controlled. Based on the closed-form expression of the derived transmission power, the optimal transmission power of admissible D2D pairs for the reuse of uplink or downlink channel resources can be obtained.

First, we consider the scenario where D2D pairs reuse the uplink channel resources of the CUs in this subsection.

Combining Inequalities (17) and (18), we obtained the minimum power limit values of $p_{m}^{u}$ and $p_{n}^{}$ as follows:

(19) $$p_{m}^{u}\geq p_{th}^{d},p_{th}^{d}=\frac{\xi_{min}^{D2D}\delta^{2}(g_{C_{n,B}}+\xi_{min}^{CUs}g_{C_{n,m}})}{g_{D_{m,m}}g_{C_{n,B}}-\xi_{min}^{D2D}\xi_{min}^{CUs}g_{D_{m,B}}g_{C_{n,m}}},$$

(20) $$p_{n}^{}\geq p_{th}^{c},p_{th}^{c}=\frac{\xi_{min}^{CUs}\delta^{2}(g_{{}_{D_{m,m}}}+\xi_{min}^{D2D}g_{D_{m,B}})}{g_{D_{m,m}}g_{C_{n,B}}-\xi_{min}^{D2D}\xi_{min}^{CUs}g_{D_{m,B}}g_{C_{n,m}}},$$

where $p_{th}^{d}$ is the minimum power limit of $p_{m}^{u}$, and $p_{th}^{c}$ is the minimum power limit of $p_{n}^{}$.

By observing Equations (11) and (20), it can be concluded that the smaller the value of $p_{n}^{}$, the higher the EE. Therefore, when the maximum value of $\eta_{ee}$ is obtained, $p_{n}^{}$ must be its minimum value pn∗, which is expressed as:

(21) Pn∗={pthc,0≤pthc≤pcmax0,pthc<0 or pthc>pcmax.

In the above equation, $p_{th}^{c}<0$ states that the obtained minimum transmission power limit of the $C_{n}$ is less than 0, which is meaningless. Hence, pn∗=0 and the corresponding D2D pair $D_{m}$ is forbidden to be included in the uplink channel resource admissible set of $C_{n}$. Similarly, $p_{th}^{c}>p_{c}^{max}$ states that the minimum transmission power limit of the $C_{n}$ is higher than the maximum limit, which is also meaningless. Hence, pn∗=0 and the corresponding D2D pair $D_{m}$ is also forbidden to be included in the uplink channel resource admissible set of $C_{n}$. Maximizing the EE of the D2D pairs must satisfy the QoS requirements of the CUs under the constraint conditions mentioned above. Otherwise, the solved pn∗ will be meaningless. When $p_{n}^{}$ is obtained from Equation (21), it is a known constant. Therefore, the optimization variables of the problem are simplified to contain only $p_{m}^{u}$ and $\chi_{m,n}^{u}$.

In this scenario, the D2D pairs do not interfere with each other since each CU’s resource can only be reused by at most one D2D pair. Hence, the optimal transmission power for each D2D pair can be obtained first. Then, the channel allocation scheme of the D2D pairs and CUs can be further solved. Assuming that the D2D pair $D_{m}$ reuses the uplink channel resource of $C_{n}$, the objective function of the optimization problem can be transformed into:

(22) ηee’=maxpmulog2(1+pmugDm,mδ2+Pn∗gCn,m)pmu+2P0.

Then, we set a variable Y as follows:

(23) Y=gDm,mδ2+pn∗gCn,m.

Therefore, Equation (22) can be expressed as:

(24) $$\eta_{ee}^{’}=\underset{p_{m}^{u}}{\max}\frac{\log_{2}(p_{m}^{u}Y+1)}{p_{m}^{u}+2P_{0}},\;p_{m}^{u}\geq 0,Y>0.$$

The optimization variable of the above formula only includes $p_{m}^{u}$. We transformed the above formula as the function $g(p_{m}^{u})$, which is given as:

(25) $$g(p_{m}^{u})=\frac{\log_{2}(p_{m}^{u}Y+1)}{p_{m}^{u}+2P_{0}},\;p_{m}^{u}\geq 0,Y>0.$$

**Proposition** **1.** *The Lambert W function can be utilized to obtain the maximum value of the function* $g(p_{m}^{u})$ *at* pmu∗=θ0−1Y *. We give the value of* $\theta_{0}$ *using Equation (26), where* W *indicates the Lambert W function:*

(26) $$\theta_{0}=\exp(W(\frac{2P_{0}Y-1}{e})+1).$$

**Proof.**  See Appendix B.

Combining Equation (26) and the maximum and minimum Constraints (15) and (19) of $p_{m}^{u}$, we obtain the optimal solution pmu∗ of the D2D pair transmission power, which is shown as follows in three different situations:

1. When $0<p_{th}^{d}\leq p_{d}^{\max}$ is established, we express the optimal solution of $p_{m}^{u}$ as:
  (27) pum∗={pthd,θ0−1Y<pthdθ0−1Y,pthd≤θ0−1Y≤pdmaxpdmax,pdmax<θ0−1Y.
2. When $p_{th}^{d}\leq 0$ is established, we express the optimal solution of $p_{m}^{u}$ as:
  (28) pum∗={pdmax,pdmax<θ0−1Yθ0−1Y,θ0−1Y≤pdmax.
3. When $p_{th}^{d}>p_{d}^{max}$ is established, we prohibit the uplink channel resource admissible set of $C_{n}$ from including the corresponding D2D pair $D_{m}$.

Consequently, according to Equations (21), (27), and (28), we obtained a solution to the transmission power control sub-problem when the D2D pairs reuse the uplink channel resources of the CUs. That is, the transmission power of $C_{n}$ is pmu∗ and the transmission power of the D2D pair $D_{m}$ is pum∗.

Similarly, when the D2D pairs reuse the downlink channel resources of the CUs, we obtained the minimum power limit of $p_{m}^{d}$ and $p_{B}^{}$ as follows through combining Inequalities (17) and (18):

(29) $$p_{m}^{d}\geq p_{th}^{d0},\;p_{th}^{d0}=\frac{\xi_{min}^{D2D}\delta^{2}(g_{C_{n,B}}+\xi_{min}^{CUs}g_{C_{B,m}^{}})}{g_{D_{m,m}}g_{C_{n,B}}-\xi_{min}^{D2D}\xi_{min}^{CUs}g_{D_{m,n}^{}}g_{C_{B,m}^{}}},$$

(30) $$p_{B}^{}\geq p_{th}^{B},\;p_{th}^{B}=\frac{\xi_{min}^{CUs}\delta^{2}(g_{{}_{D_{m,m}}}+\xi_{min}^{D2D}g_{D_{m,n}^{}})}{g_{D_{m,m}}g_{C_{n,B}}-\xi_{min}^{D2D}\xi_{min}^{CUs}g_{D_{m,n}^{}}g_{C_{B,m}^{}}},$$

where $p_{th}^{d0}$ is the minimum power limit of $p_{m}^{d}$, and $p_{th}^{B}$ is the minimum power limit of $p_{B}^{}$.

By observing Equations (11) and (30), it can be concluded that the smaller the value of $p_{B}^{}$, the higher the EE. Therefore, when the maximum value of $\eta_{ee}$ is obtained, $p_{B}^{}$ must be its minimum value pB∗, which is expressed by:

(31) pB∗={pthB,0≤pthB≤pBmax0,pthB<0 or pthB>pBmax.

Then, we set the variables Z and $\theta_{1}$ as follows:

(32) Z=gDm, mδ2+pB∗gCB, m,

(33) $$\theta_{1}=\exp(W(\frac{2P_{0}Z-1}{e})+1).$$

Combining Equation (33) and the maximum and minimum Constraints (15) and (29) of $p_{m}^{d}$, we obtained the optimal solution Pmd∗ of the D2D pair transmission power, which is shown as follows in three different situations:

1. When $0<p_{th}^{d0}\leq p_{d}^{max}$ is established, we express the optimal solution of $p_{m}^{d}$ as:
  (34) pmd∗={pthd0,θ1−1Z<pthd0θ1−1Z,pthd0≤θ1−1Z≤pdmaxpdmax,pdmax<θ1−1Z.
2. When $p_{th}^{d0}\leq 0$ is established, we express the optimal solution of $p_{m}^{d}$ as:
  (35) pmd∗={pdmax,pdmax<θ1−1Zθ1−1Z,θ1−1Z≤pdmax.
3. When $p_{th}^{d0}>p_{d}^{max}$ is established, we prohibit the downlink channel resource admissible set of $C_{n}$ from including the corresponding D2D pair $D_{m}$.

As a consequence, according to Equations (31), (34), and (35), we obtained a solution to the transmission power control sub-problem when the D2D pairs reuse the downlink channel resources of the CUs. That is, the transmission power of the BS corresponding to the D2D pair $D_{m}$ is pB∗, and the transmission power of the D2D pair $D_{m}$ is pmd∗.

As mentioned above, to maximize the EE of the D2D pairs while satisfying the QoS, it is forbidden to include unqualified D2D pairs into the set of admissible channel resources. According to the transmission power allocation process of the CUs and D2D pairs in the network, D2D pairs that do not meet the requirements are composed of the following six categories:

1. If $p_{th}^{c}<0$, i.e., the minimum transmission power limit of $C_{n}$ is less than 0, this cannot meet the minimum SINR requirements of the CUs. The corresponding D2D pair $D_{m}$ is prevented from reusing the uplink channel resource of $C_{n}$.
2. If $p_{th}^{B}<0$, i.e., the minimum transmission power limit of BS is less than 0, this cannot meet the minimum SINR requirement of the CUs. Similar to the previous category, the corresponding D2D pair $D_{m}$ is prevented from reusing the downlink channel resource of $C_{n}$.
3. If $p_{th}^{c}>p_{c}^{max}$, i.e., the minimum transmission power limit of $C_{n}$ is higher than the maximum limit, to meet the QoS requirements of the CUs within the maximum transmission power, the corresponding D2D pair $D_{m}$ is prevented from reusing the uplink channel resource of $C_{n}$.
4. If $p_{th}^{B}>p_{B}^{max}$, i.e., the minimum transmission power limit of BS is higher than the maximum limit, the corresponding D2D pair $D_{m}$ is prevented from reusing the downlink channel resource of $C_{n}$, similar to the last category.
5. If $p_{th}^{d}>p_{d}^{max}$, i.e., the minimum transmission power limit of D2D pair $D_{m}$ reusing uplink channel is higher than the maximum limit, the optimization process must be performed under the requirements of the minimum SINR and the maximum transmission power of the D2D pair $D_{m}$. Otherwise, the optimization results will be meaningless. Therefore, the corresponding D2D pair $D_{m}$ is prevented from reusing the uplink channel resource of $C_{n}$.
6. If $p_{th}^{d0}>p_{d}^{max}$, i.e., the minimum transmission power limit of D2D pair $D_{m}$ reusing downlink channel is higher than the maximum limit, the corresponding D2D pair $D_{m}$ is prevented from reusing the downlink channel resource of $C_{n}$, similar to the previous category.

### 3.2. Channel Allocation

This section focuses on the second sub-problem. To maximize the overall EE of the D2D pairs, we utilized the Kuhn–Munkres algorithm to allocate channel resources to the D2D pairs that have been allocated power reasonably. Each D2D pair will choose its reusing mode according to the EE of its different reusing mode.

Combining the transmission power (pn∗ , pB∗ , pmd∗ , pmu∗) obtained as the solution to sub-problem 1, the channel allocation problem can be expressed as Equations (36) and (37). The optimization objective was to maximize the achievable EE of the D2D pairs with the optimal value of the reusing mode $\left(\chi_{m,n}^{u},\;\chi_{m,n}^{d}\right)$:

(36) ηee=maxχm,nd, χm,nu∑∑m∑n[χm,ndRm,nd∗+χm,nuRm,nu∗]∑m∑n[χm,ndpmd∗+χm,nupmu∗]+∑m=1M2P0,

(37) $$\begin{array}{l} \sum_{m}[\chi_{m,n}^{u}+\chi_{m,n}^{d}]\leq 1,\;\chi_{m,n}^{u},\;\chi_{m,n}^{d}\in \{0,1\},\forall D_{m}\in D_{A},\forall C_{n}\in C\\ \sum_{n}[\chi_{m,n}^{u}+\chi_{m,n}^{d}]\leq 1,\chi_{m,n}^{u},\chi_{m,n}^{d}\in \{0,1\},\;\forall D_{m}\in D_{A},\;\forall C_{n}\in C \end{array}$$

In Equation (36), Rm,nu∗ is the SE of the D2D pair $D_{m}$ reusing the uplink channel resource of $C_{n}$:

(38) Rm,nu∗=log2(1+pmu∗gDm,mδ2+pn∗gCn,m),∀Dm∈DA,∀Cn∈C,

while Rm,nd∗ is the SE of the D2D pair $D_{m}$ reusing the downlink channel resource of $C_{n}$:

(39) Rm,nd∗=log2(1+pmd∗gDm,mδ2+p∗BgCB,m),∀Dm∈DA,∀Cn∈C,

where $D_{A}$ represents a set of admissible D2D pairs and C represents a set of CUs. Based on either an uplink or downlink resource being assigned for reuse, the EE of the D2D pairs are calculated using the power control results, which are shown as follows:

(40) ηm,nu∗=log2(pmu∗Y+1)pmu∗2P0,

(41) ηm,nd∗=log2(pmd∗Z+1)pmd∗+2P0,

where ηm,nu∗ is the EE of the D2D pair $D_{m}$ reusing the uplink channel resource of $C_{n}$ and ηm,nd∗ is the EE of the D2D pair $D_{m}$ reusing the downlink channel resource of $C_{n}$. Therefore, ηm,nu∗ and ηm,nd∗ are used to represent the EE weights of the D2D pairs reusing the uplink and downlink channel resources, respectively.

Based on Equation (36), we considered the channel allocation problem through a graph model and formulated it as a weighted bipartite-graph-matching problem in graph theory. The goal of optimizing the overall EE of the D2D pairs was transformed into maximizing the total weight of the constructed graph, namely the maximum weight-matching of the bipartite graph. The vertices in the graph denoted the CU’s uplink–downlink resources and the D2D pairs, and the edge weights represented the EE weights of the D2D pairs when the CUs share their channels with D2D pairs.

We built the D2D pair’s EE matrix H based on the above graph model:

(42) H=[η1,1u∗η1,2u∗⋯η1,Nu∗η2,1u∗η2,2u∗⋯η2,Nd∗⋮⋮⋮ηM,1u∗ηM,2u∗⋯ηM,Nu∗η1,1d∗η1,2d∗⋯η1,Nd∗η2,1d∗η2,2d∗⋯η2,Nd∗⋮⋮⋮ηM,1d∗ηM,2d∗⋯ηM,Nd∗].

When the D2D pair $D_{m}$ is allowed to reuse the resource of $C_{n}$, they establish a connection and use ηm,nu∗ or ηm,nd∗ as the weight.

The above matching problem can be solved using the Kuhn–Munkres algorithm in Edmonds and Karp [36], where the details are beyond the scope of this study. The computational complexity of the Kuhn–Munkres algorithm is $o(N^{3})$, which can solve the matching result of the entire network in polynomial time.

In summary, the resource allocation problem of the cellular and D2D hybrid network was decomposed into two sub-problems, namely optimal power control and channel allocation for the D2D pairs. First, to maximize the EE of each D2D pair while meeting the QoS requirements, the optimal transmission power of the D2D pairs reusing the uplink and downlink channel resources were respectively derived based on the Lambert W function. Then, to maximize the overall EE of the D2D pairs, a bipartite graph was constructed for the set of admissible D2D pairs and the corresponding CUs. The Kuhn–Munkres algorithm was used to obtain the channel-matching result.

Compared with existing algorithms, we provide a simple analysis of the tradeoff between the EE performance and computational complexity. In terms of power control, although the fixed transmission power allocation method used in Zulhasnine et al. [24] has lower computational complexity, it has lower flexibility and ignores the QoS requirements of the D2D pairs and CUs. The computational complexity of the power control method proposed in this study depends on the closed-form expression of the transmission power. While guaranteeing the SINR of the CUs, it effectively improves the EE performance of the D2D pairs.

In terms of channel allocation, the computational complexity of the heuristic channel allocation scheme in Zulhasnine et al. [24] is $o(N^{2})$. However, it was considered from the perspective of local optimization. Furthermore, it only briefly discusses the interference information between users and ignores the power collaboration between the D2D pairs and CUs. Based on game theory, the computational complexity of the channel allocation in References [30,31] is related to the number of iterations and the suboptimal solution or the optimal solution is ultimately obtained. The channel allocation proposed in this study can obtain the optimal channel-matching scheme through the Kuhn–Munkres algorithm with complexity $o(N^{3})$.

The resource allocation algorithm that combines the uplink–downlink channel resources is summarized in Algorithm 1.
**Algorithm 1:** Resource Allocation Algorithm That Combines the Uplink–Downlink Resources to Maximize Energy EfficiencyStep 1: Initialize1:  $D_{A}⇐D$;Step 2: Power Control2:  for $C_{n}\in C$, $D_{m}\in D_{A}$ do3:    Calculate the minimum transmission power limit of the CUs, D2D pairs, and BS according to Constraints (19), (20), (29), and (30);4:    Calculate the transmission power of the CUs and BS based on Equations (21) and (31);5:    Calculate the optimal transmission power of the D2D pairs reusing the uplink or downlink channel resources based on Equations (27), (28), (34), and (35);6:    if $\;p_{th}^{c}<0$, $p_{th}^{c}>p_{c}^{\max}$, or $p_{th}^{d}>p_{d}^{\max}$ then7:     Prevent the D2D pair $D_{m}$ in the admissible set $D_{A}$ from reusing the uplink channel resource of $C_{n}$;8:    end if9:    if $\;p_{th}^{B}<0$, $p_{th}^{B}>p_{B}^{max}$, or $p_{th}^{d0}>p_{d}^{max}$ then10:     Prevent the D2D pair $D_{m}$ in the admissible set $D_{A}$ from reusing the downlink channel resource of $C_{n}$;11:    end if12:  end forStep 3: Channel Allocation13:  Obtain the channel allocation set $Χ=\left\{\chi_{m,n}^{u},\chi_{m,n}^{d}\right\}$ based on the Kuhn–Munkres algorithm.

## 4. Numerical Results

### 4.1. Simulation Design

In this section, the EE of the system and the EE of the D2D pairs are selected as the algorithm performance evaluation indicators. The system EE refers to the sum of the EE, including all the D2D pairs’ EEs and the CUs’ EEs in the network. Since the power consumption of the BS, which is powered by external power, it is not considered in this study. The EE of the system is given by:

(43) $$\eta_{\mathrm{sum}}=\frac{R_{\mathrm{sum}}}{\sum_{m=1}^{M}\left[\sum_{n=1}^{N}\left(\chi_{m,n}^{d}p_{m}^{d}+\chi_{m,n}^{u}p_{m}^{u}\right)+2P_{0}\right]+\sum_{n=1}^{N}\left[\sum_{m=1}^{M}\left(\chi_{m,n}^{d}p_{B}^{}+\chi_{m,n}^{u}p_{n}^{}\right)+P_{0}\right]},$$

where $p_{n}^{}$ and $p_{B}^{}$ indicate the transmission power of $C_{n}$ and the BS, respectively. N is the number of CUs. $p_{m}^{u}$ and $p_{m}^{d}$ indicate the transmission power of the D2D pair $D_{m}$ reusing the uplink channel and downlink channel, respectively. M is the number of D2D pairs. $\chi_{m,n}^{u}$ and $\chi_{m,n}^{d}$ are the identifiers of the resource reuse. $R_{\mathrm{sum}}$ refers to the total sum of all CUs and D2D pairs accessing the network.

The algorithm proposed in this paper, labeled as “proposed” below, is compared with the following three algorithms.

1. Heuristic algorithm reusing the uplink spectrum resources [24]: The basic principle of this algorithm is that the BS preferentially selects the cellular link with a high channel gain and the D2D communication link with the least interference to reuse the same channel. The algorithm consists of access control based on interference control, fixed power allocation, and heuristic channel allocation. This algorithm is feasible and straightforward, and the interference caused by the D2D link to the cellular link is small. However, the power between the D2D pairs and CUs are not considered for coordination; meanwhile, the algorithm is based on the assumption that only uplink resources can be shared. Therefore, the performance of D2D communication is not sufficiently improved. The algorithm is labeled “HeuristicOU.”
2. Heuristic algorithm reusing the downlink spectrum resources [24]: The principle of this algorithm is similar to the “HeuristicOU” algorithm, where the difference lies in the assumption that only downlink resources can be shared. The algorithm is labeled “HeuristicOD.”
3. Stable matching algorithm reusing the uplink spectrum resources [30]: This algorithm allocates optimal transmission power to the D2D pairs. Then, the channel gain ratio of the communication link and the interference link is defined as the sequence value of the user-matching preference. The Gale–Shapley algorithm is utilized to establish the preferences of each user equipment and complete the matching of the D2D pairs and CUs. This algorithm effectively improves the EE of the D2D pairs. However, it does not jointly reuse the uplink and downlink spectrum resources, and its channel-matching algorithm only obtains stable matching results. Therefore, the EE of the D2D pairs in the network could still be further improved. The algorithm is labeled “GaSaBa.”

Using the MATLAB platform (R2019b, developed by MathWorks), we assumed a single cell with a radius of 250 m, and the CUs and D2D pairs were distributed randomly in the cell. The average simulation result is based on 1000 iterations. The values of the simulation parameters were based on References [8,33] and are summarized in Table 1**.**

### 4.2. Results and Discussions

#### 4.2.1. Effect of the D2D Transmission Distance on the System Performance

**Figure 2:**
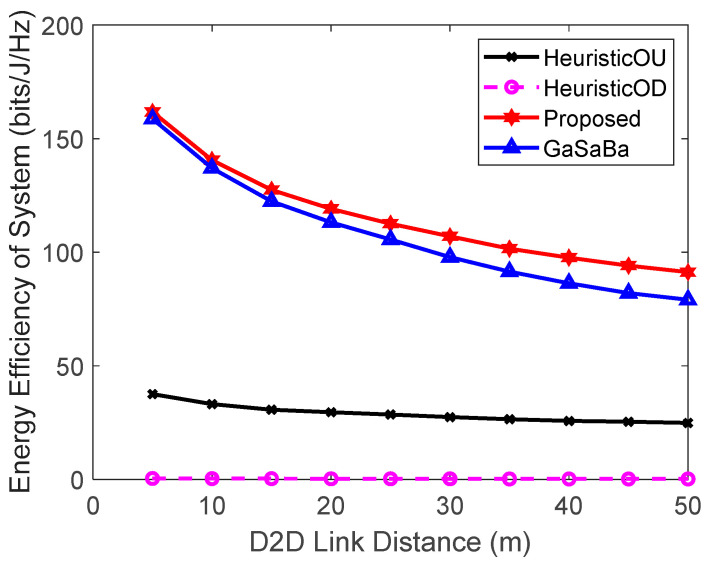
Relationship between the energy efficiency (EE) of the system and the D2D link distance.

**Figure 3:**
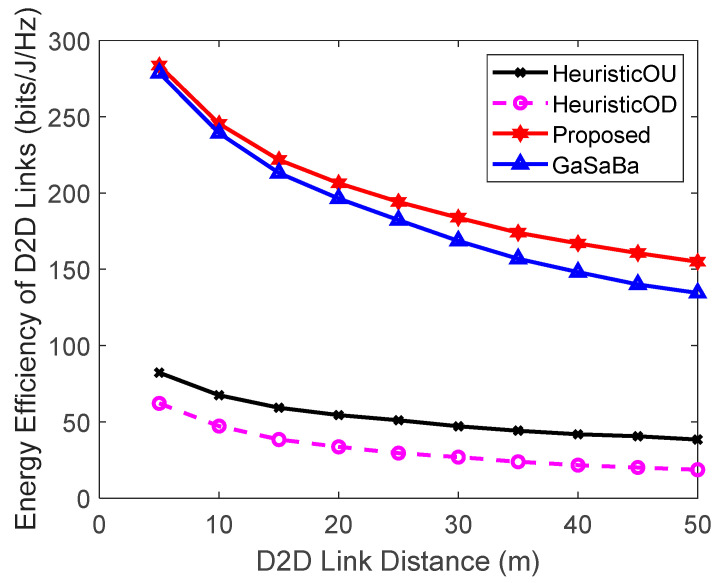
Relationship between the EE of D2D links and the D2D link distance.

Figure 2 plots the total EE of the system versus $L_{d}$ and Figure 3 plots the EE of the D2D pairs versus $L_{d}$, where M=6 D2D pairs and N=10 CUs. Although the EE of the four algorithms decreased with the increase of the communication distance, the system EE and the D2D pair’s EE of the proposed algorithm were higher than those of the other three algorithms in the whole regime.

As $L_{d}$ increased, compared to the short-distance scenario, a higher transmission power was required to meet the same QoS requirements. However, increasing the transmission power brought about more interference and power consumption, resulting in an EE loss that could not be compensated for by the corresponding SE gain. Therefore, the EE decreased as the communication distance increased. The proposed algorithm and the GaSaBa algorithm allocated optimal transmission power to the D2D pairs based on maximizing EE, with the QoS constraints of both the CUs and D2D pairs in mind. Furthermore, the proposed algorithm jointly reused the uplink–downlink spectrum resources, and its channel allocation further maximized the EE of the D2D pairs. Hence, the EE performance was better than the other three algorithms. The HeuristicOU and HeuristicOD algorithms only reused the uplink or downlink spectrum resources with constant transmission power. Furthermore, since their channel matching algorithms were performed based on the channel gains, the SE losses increased when the channel gains decreased. For these reasons, the EE performance of the HeuristicOD algorithm, which was assigned the fixed transmission power of the BS, always performed the worst among the four algorithms.

#### 4.2.2. Effect of Number of D2D Links on the System Performance

**Figure 4:**
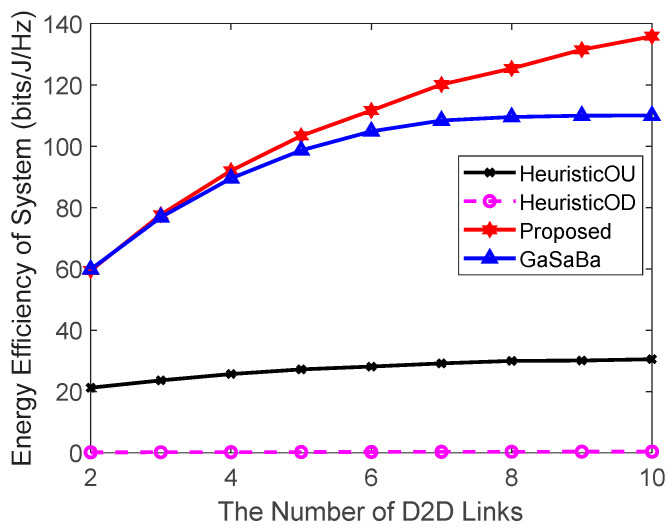
Relationship between the EE of the system and the number of D2D links.

**Figure 5:**
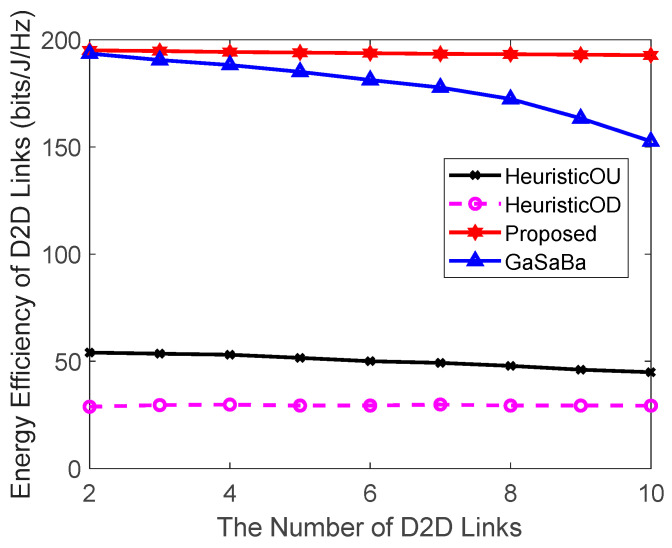
Relationship between the EE of the D2D links and the number of D2D links.

Figure 4 plots the total EE of the system versus the number of D2D links and Figure 5 plots the EE of the D2D pairs versus the number of D2D links, where $L_{d}=25\;m$ and N=10 CUs. The simulation results demonstrate that the system EE and D2D pair’s EE of the proposed algorithm were higher than the other three algorithms. Figure 4 shows that the system EE using the four algorithms increased at different rates. As the number of D2D pairs increased, the number of reused CUs also increased. Hence, the EE of the CUs became higher, which in turn increased the EE of the system. The HeuristicOD algorithm had the slowest EE growth rate, and the HeuristicOU algorithm was slightly better. The reason for this was that the two algorithms transmitted data in a fixed power allocation mode, which limited the improvement of EE. The GaSaBa algorithm, which reused the uplink resources with a stable channel-matching approach, significantly reduced the system EE growth rate when the number of D2D links was close to the number of CUs. This was because, as the number of D2D links increased, each D2D pair found it more difficult to match with a better partner in a limited matching market. Therefore, the system EE could not be further maximized. Different from these three, the proposed algorithm jointly reused the uplink–downlink resources and obtained more available channel resources than the other three comparison algorithms. Hence, it could take full advantage of the increased total number of available orthogonal channels and exploit more benefits from the diversity of choices, thereby achieving a better EE performance.

Figure 5 shows that the EE of the D2D pairs when using each of the four algorithms decreased slightly as the number of D2D pairs increased, and the performance of the proposed algorithm decreased the slowest. The reason for this was that in the other three algorithms, as the number of D2D pairs increases, the number of available channel resources decreased. Hence, it was more difficult for the D2D pairs to match the channel resources of the lower-interference CUs. The proposed algorithm combined the uplink–downlink spectrum resources, guaranteeing the better stability of the EE of the D2D pairs as the number of D2D links increased.

#### 4.2.3. Effect of Threshold of the CUs SINR on System Performance

**Figure 6:**
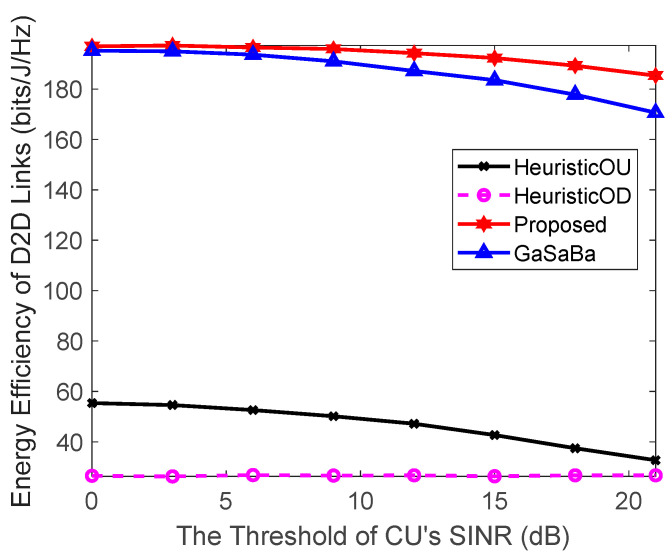
Relationship between the EE of the D2D links and the threshold of the CU’s signal-to-interference-plus-noise ratio (SINR).

**Figure 7:**
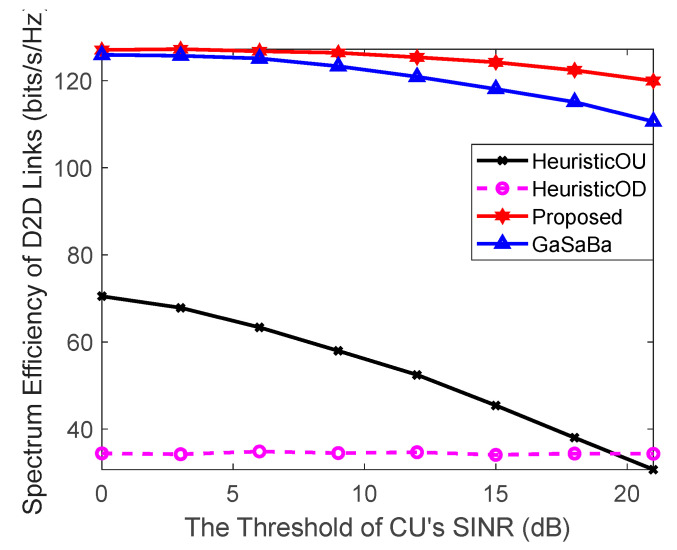
Relationship between the SE of the D2D links and the threshold of the CU’s SINR.

Figure 6 plots the EE of the D2D pairs versus the threshold of the CUs’ SINR and Figure 7 plots the SE of the D2D pairs versus the threshold of the CUs’ SINR, where M=6 D2D pairs, N=10 CUs, and $L_{d}=25\;m$. As can be seen from Figure 6, the performance of the proposed algorithm was superior to the other three algorithms. With the increase of the threshold of the CUs’ SINR, the D2D pair’s EE of each of the four algorithms decreased. Because of the QoS constraints of the CUs, the performance of the D2D pair’s SE was gradually sacrificed. Furthermore, the EE and SE performances of the HeuristicOD algorithm were the worst among the four algorithms due to the high fixed power allocation.

In Figure 7, we can see that with the increase of the threshold of the CU’s SINR, the D2D pair’s SE of the proposed algorithm was still higher than the other three algorithms. The SE of the HeuristicOU algorithm decreased rapidly and the SE of the other three algorithms decreased slowly. To guarantee the performance requirements of the CUs, the power allocation of the proposed algorithm was optimized under the premise of meeting the minimum SINR requirements of the CUs; therefore, the results of the optimization were partially affected by the threshold of the CU’s SINR.

Through a comprehensive analysis of the above simulation results, the proposed algorithm achieved the best performance regarding the EE of the D2D pairs, the SE of the D2D pairs, and the EE of the system. The reason for this was that due to the uplink–downlink channel reuse, the proposed algorithm optimized the individual D2D’s EE to obtain the optimal power allocation, which effectively improved the EE of the D2D pairs. Furthermore, the channel allocation algorithm was used to obtain optimal channel-resource matching, which maximized the achievable EE of the D2D pairs. The GaSaBa algorithm did not jointly reuse the uplink–downlink spectrum resources, and its channel matching algorithm only obtained stable matching results. Therefore, the performance of D2D pairs in the network could still be further improved. The other two algorithms, namely HeuristicOU and HeuristicOD algorithms, only reused the uplink or downlink spectrum resources with constant transmission power. Moreover, the power between the D2D pairs and CUs were not considered for coordination, resulting in the performance of the D2D communication not being sufficiently improved. Therefore, the EE performance of these three algorithms was lower than the proposed algorithm. Moreover, the proposed algorithm provides an idea for research directions regarding resource allocation in green D2D communication. Future works include implementing many-to-one and many-to-many matching, and combining specific applications of the IoT.

## 5. Conclusions

In this paper, a two-layer, energy-efficient algorithm was proposed for the resource allocation problem in D2D communications. Taking into account reusing uplink–downlink resources, we formulated a one-to-one matching problem to maximize the achievable EE of the D2D pairs under maximum transmission power and QoS constraints. To solve the NP-hard problem, we divided the original problem into two sub-problems: power control and channel allocation. First, by satisfying the conditions of the minimum SINR of the CUs and D2D pairs, the closed-form expression of power allocation was solved through the Lambert W function. By assigning either an uplink or downlink resource to reuse, the EE of each D2D pair was calculated using the power control results. The channel allocation scheme based on the Kuhn–Munkres algorithm utilized EE weights to optimize the overall EE of the D2D pairs. Extensive simulation results showed that compared with the existing solutions, the proposed algorithm remarkably improved the system EE and the D2D pair’s EE under the premise of ensuring the performance of the CUs.

## Figures and Tables

**Table 1:** Simulation parameters.

Parameters	Value
Cell radius	250 m
Noise power spectral density	−174 dBm/Hz
CU’s maximum transmission power $p_{c}^{max}$	24 dBm
D2D’s maximum transmission power $p_{d}^{max}$	21 dBm
BS’s maximum transmission power $p_{B}^{max}$	46 dBm
CU’s SINR thresholds $\xi_{min}^{CUs}$	U~[0,25] dB
D2D’s SINR thresholds $\xi_{min}^{D2D}$	U~[0,25] dB
D2D’s maximum transmission distance $L_{d}$	25, [5, 10, …, 50] m
Number of CUs	10
Number of D2D pairs	6, 2–10
Multipath fading parameters $\beta_{n,m}$ (mean of exponential distribution)	1
Shadow fading $\lambda_{n,m}$ (standard deviation of log-normal distribution)	8 dB
Path loss factor α	4
Equipment circuit loss $P_{0}$	50 mW

## Appendix A

**Table A1:** Notation list.

Notations	Variables and Parameters
$C=\{C_{1},...,C_{n},...,C_{N}\}$	CUs
$D=\{D_{1},...,D_{m},...,D_{M}\}$	D2D pairs
$f_{n}^{u}$	uplink resource of $C_{n}$
$f_{n}^{d}$	downlink resource of $C_{n}$
$D_{1}\_\mathrm{Tx}$	D2D transmitter of $D_{1}$
$D_{1}\_\mathrm{Rx}$	D2D receiver of $D_{1}$
$g_{C_{n.m}}$	interference channel gain from $C_{n}$ to the D2D receiver of $D_{m}$
K	pathloss constant
$\beta_{n,m}$	multipath fading parameter from $C_{n}$ to the D2D receiver of $D_{m}$
$\lambda_{n,m}$	shadow gain from $C_{n}$ to the receiver of $D_{m}$
$d_{n,m}$	distance from $C_{n}$ to the receiver of $D_{m}$
α	path loss factor
$g_{D_{m,m}}$	channel gain of $D_{m}$
$g_{C_{n,B}}$	channel gain between $C_{n}$ and BS
$g_{D_{m,B}}$	interference channel gain from the D2D transmitter of $D_{m}$ to the BS
$g_{C_{B,m}^{}}$	interference channel gain from the BS to the D2D receiver of $D_{m}$
$g_{D_{m,n}^{}}$	interference channel gain from the D2D transmitter of $D_{m}$ to $C_{n}$
$z_{n}^{rx}$	received signal at the BS
$p_{n}^{}$	transmission power of $C_{n}$
$p_{m}^{u}$	transmission power of $D_{m}$ reusing an uplink channel
$x_{n}$	transmission signal of $C_{n}$
$y_{m}$	transmission signal of $D_{m}$
$\zeta_{n}$	noise in each channel
$\chi_{m,n}^{u}$	binary variable that the uplink resource of $C_{n}$ allocated to $D_{m}$
$\gamma_{n}^{u}$	SINR at the BS
$y_{m}^{rx}$	received signal at the D2D pair $D_{m}$ reusing an uplink channel
$\gamma_{m}^{u}$	SINR at the receiver of D2D pair $D_{m}$ reusing an uplink channel
$\gamma_{n}^{d}$	SINR of the receiver of $C_{n}$
$\gamma_{m}^{d}$	SINR at the receiver of the D2D pair $D_{m}$ reusing a downlink channel
$p_{B}^{}$	transmission power of the BS
$p_{m}^{d}$	transmission power of the $D_{m}$ reusing a downlink channel
$\chi_{m,n}^{d}$	binary variable that the downlink resource of $C_{n}$ allocated to $D_{m}$
$R_{m,n}^{u}$	SE of the $D_{m}$ reusing an uplink channel of $C_{n}$
$R_{m,n}^{d}$	SE of the $D_{m}$ reusing a downlink channel of $C_{n}$
$P_{0}$	equipment circuit loss
$\eta_{ee}$	total EE of the D2D pairs
$p_{c}^{max}$	CU’s maximum transmission power
$p_{d}^{max}$	D2D’s maximum transmission power
$p_{B}^{max}$	BS’s maximum transmission power
$\xi_{min}^{CUs}$	CU’s SINR thresholds
$\xi_{min}^{D2D}$	D2D’s SINR thresholds
$L_{d}$	D2D’s maximum transmission distance

## Appendix B

According to Equations (23) and (25), we set $\theta =p_{m}^{u}Y+1$, where it can be seen that θ>1; furthermore, $p_{m}^{u}=\frac{\theta -1}{Y}$. Therefore, we transformed Equation (25) into the function g(θ) given as:

(A1) $$g(\theta )=\frac{Y\ln(\theta )}{(\theta -1+2P_{0}Y)\cdot \ln2}.$$

We evaluated the derivative of the variable θ in Equation (A1) to obtain the expression (A2). Then, we set its derivative to be equal to zero, namely $\frac{\partial g(\theta )}{\partial \theta }=0$:

(A2) $$\frac{\partial g(\theta )}{\partial \theta }=\frac{\theta -1+2P_{0}Y-\theta \ln\theta }{\theta {\left((\theta -1+2P_{0}Y)\ln2\right)}^{2}}\cdot Y\ln2.$$

We set $f(\theta )=\theta -1+2P_{0}Y-\theta \ln\theta$, and differentiate f(θ) to obtain the derivative $\frac{\partial f(\theta )}{\partial \theta }=-\ln\theta$. Since θ>1, the derivative $\frac{\partial f(\theta )}{\partial \theta }<0$ is established and f(θ) decreases monotonically in the interval θ∈(1,+∞). Additionally, if θ→+∞, f(θ)<0. If θ→1, f(θ)>0. Hence, there must exist $\theta =\theta_{0}$ to produce $f(\theta_{0})=0$, i.e., the equality $\frac{\partial g(\theta )}{\partial \theta }=0$ has a solution. As a consequence, there must exist pmu*=θ0−1Y that ensures that $g(p_{m}^{u})$ increases monotonically in the interval (0,pmu*) while decreasing monotonically in the interval (pmu*,+∞). In other words, $g(p_{m}^{u})$ has its maximum value at pmu*=θ0−1Y.

Then, we let $f(\theta =\theta_{0})=0$, i.e., Equation (A3) as follows:

(A3) $$\theta_{0}\ln\theta_{0}-\theta_{0}=2P_{0}Y-1.$$

Then, Equation (A3) is transformed into $\theta_{0}\left(\ln\theta_{0}-1\right)=2P_{0}Y-1$, which is equivalent to the following Equation (A4):

(A4) $$\frac{\theta_{0}}{e}\ln\frac{\theta_{0}}{e}=\frac{2P_{0}Y-1}{e}.$$

Due to $2P_{0}Y>0$, the right side of the equality $\frac{2P_{0}Y-1}{e}>-1$ is established, which meets the requirements of the Lambert W function. As a result, through the Lambert W function, we obtained the specific value $\theta_{0}$ represented by the following expression:

(A5) $$\theta_{0}=\exp(W(\frac{2P_{0}Y-1}{e})+1).$$

Equation (A5) is also shown in Equation (26) and the optimal solution pmu*=θ0−1Y of the function $g(p_{m}^{u})$ can be further obtained.

At this point, we have completed the proof.

## References

[1] Ben-Daya M., Hassini E., Bahroun Z. (2019). Internet of things and supply chain management: A literature review. Int. J. Prod. Res..

[2] Yu B., Zhang X., Palmieri F., Creignou E., You I. (2019). A deep learning approach for maximum activity links in D2D communications. Sensors.

[3] Nitti M., Stelea G.A., Popescu V., Fadda M. (2019). When social networks meet D2D communications: A survey. Sensors.

[4] Chang W., Teng J.-C. (2018). Energy efficient relay matching with bottleneck effect elimination power adjusting for full-duplex relay assisted D2D networks using mmWave technology. IEEE Access.

[5] Gandotra P., Jha R.K., Jain S. (2018). Sector-based radio resource allocation (SBRRA) algorithm for better quality of service and experience in device-to-device (D2D) communication. IEEE Trans. Veh. Technol..

[6] Fodor G., Dahlman E., Mildh G., Parkvall S., Reider N., Miklós G., Turányi Z. (2012). Design aspects of network assisted device-to-device communications. IEEE Commun. Mag..

[7] Liu J., Kawamoto Y., Nishiyama H., Kato N., Kadowaki N. (2014). Device-to-device communications achieve efficient load balancing in LTE-advanced networks. IEEE Wirel. Commun..

[8] Li R., Hong P., Xue K., Zhang M., Yang T. (2020). Energy-Efficient Resource Allocation for High-Rate Underlay D2D Communications with Statistical CSI: A One-to-Many Strategy. IEEE Trans. Veh. Technol..

[9] Lei L., Kuang Y., Cheng N., Shen X.S., Zhong Z., Lin C. (2015). Delay-optimal dynamic mode selection and resource allocation in device-to-device communications—Part I: Optimal policy. IEEE Trans. Veh. Technol..

[10] Liu J., Nishiyama H., Kato N., Guo J. (2015). On the outage probability of device-to-device-communication-enabled multichannel cellular networks: An RSS-threshold-based perspective. IEEE J. Sel. Areas Commun..

[11] Liu J., Kato N., Ma J., Kadowaki N. (2014). Device-to-device communication in LTE-advanced networks: A survey. IEEE Commun. Surv. Tutor..

[12] Golrezaei N., Mansourifard P., Molisch A.F., Dimakis A.G. (2014). Base-station assisted device-to-device communications for high-throughput wireless video networks. IEEE Trans. Wirel. Commun..

[13] Kai C., Li H., Xu L., Li Y., Jiang T. (2018). Energy-efficient device-to-device communications for green smart cities. IEEE Trans. Ind. Inform..

[14] Jiang L., Tian H., Xing Z., Wang K., Zhang K., Maharjan S., Gjessing S., Zhang Y. (2016). Social-aware energy harvesting device-to-device communications in 5G networks. IEEE Wirel. Commun..

[15] Chen Y., Xie G., Li R. (2018). Reducing energy consumption with cost budget using available budget preassignment in heterogeneous cloud computing systems. IEEE Access.

[16] Xie G., Jiang J., Liu Y., Li R., Li K. (2017). Minimizing energy consumption of real-time parallel applications using downward and upward approaches on heterogeneous systems. IEEE Trans. Ind. Inform..

[17] Zhang Z., Wang L., Zhang J. (2017). Energy efficiency of D2D multi-user cooperation. Sensors.

[18] Zhou L., Wu D., Chen J., Dong Z. (2017). Greening the smart cities: Energy-efficient massive content delivery via D2D communications. IEEE Trans. Ind. Inform..

[19] Wang F., Xu C., Song L., Han Z. (2014). Energy-efficient resource allocation for device-to-device underlay communication. IEEE Trans. Wirel. Commun..

[20] Chen B., Yang C., Wang G. (2017). High-throughput opportunistic cooperative device-to-device communications with caching. IEEE Trans. Veh. Technol..

[21] Jiang Y., Liu Q., Zheng F., Gao X., You X. (2015). Energy-efficient joint resource allocation and power control for D2D communications. IEEE Trans. Veh. Technol..

[22] Wei L., Hu R.Q., Qian Y., Wu G. (2015). Energy efficiency and spectrum efficiency of multihop device-to-device communications underlaying cellular networks. IEEE Trans. Veh. Technol..

[23] Li Y., Zhang Z., Wang H., Yang Q. (2018). SERS: Social-aware energy-efficient relay selection in D2D communications. IEEE Trans. Veh. Technol..

[24] Zulhasnine M., Huang C., Srinivasan A. Efficient resource allocation for device-to-device communication underlaying LTE network. Proceedings of the 2010 IEEE 6th International Conference on Wireless and Mobile Computing, Networking and Communications.

[25] Li Y., Jin D., Yuan J., Han Z. (2014). Coalitional games for resource allocation in the device-to-device uplink underlaying cellular networks. IEEE Trans. Wirel. Commun..

[26] Hoang T.D., Le L.B., Le-Ngoc T. (2016). Resource allocation for D2D communication underlaid cellular networks using graph-based approach. IEEE Trans. Wirel. Commun..

[27] Kaufman B., Lilleberg J., Aazhang B. (2013). Spectrum sharing scheme between cellular users and ad-hoc device-to-device users. IEEE Trans. Wirel. Commun..

[28] Wang Q., Wang W., Jin S., Zhu H., Zhang N.T. (2014). Quality-optimized joint source selection and power control for wireless multimedia D2D communication using Stackelberg game. IEEE Trans. Veh. Technol..

[29] Rahman M.A., Lee Y., Koo I. (2018). Energy-efficient power allocation and relay selection schemes for relay-assisted d2d communications in 5g wireless networks. Sensors.

[30] Liu S., Wu Y., Li L., Liu X., Xu W. (2019). A two-stage energy-efficient approach for joint power control and channel allocation in D2D communication. IEEE Access.

[31] Zhou Z., Ota K., Dong M., Xu C. (2016). Energy-efficient matching for resource allocation in D2D enabled cellular networks. IEEE Trans. Veh. Technol..

[32] Zhou Z., Ma G., Dong M., Ota K., Xu C., Jia Y. (2016). Iterative energy-efficient stable matching approach for context-aware resource allocation in D2D communications. IEEE Access.

[33] Zhang R., Cheng X., Yang L., Jiao B. (2014). Interference graph-based resource allocation (InGRA) for D2D communications underlaying cellular networks. IEEE Trans. Veh. Technol..

[34] Phunchongharn P., Hossain E., Kim D.I. (2013). Resource allocation for device-to-device communications underlaying LTE-advanced networks. IEEE Wirel. Commun..

[35] Corless R.M., Gonnet G.H., Hare D.E., Jeffrey D.J., Knuth D.E. (1996). On the Lambert W function. Adv. Comput. Math..

[36] Edmonds J., Karp R.M. (1972). Theoretical improvements in algorithmic efficiency for network flow problems. J. ACM (JACM).

[37] Kwon H., Birdsall T. (1986). Channel capacity in bits per joule. IEEE J. Ocean. Eng..

